# 4,7-Diaza-1-azoniacyclo­nonane bromide

**DOI:** 10.1107/S1600536809024313

**Published:** 2009-07-01

**Authors:** Thorsten Allscher, Peter Klüfers, Christine Neumann

**Affiliations:** a Ludwig-Maximilians-Universität, Department Chemie und Biochemie, Butenandtstrasse 5–13, 81377 München, Germany

## Abstract

The title compound, C_6_H_16_N_3_^+^·Br^−^, is the bromide of the monoprotonated aza­macrocyclic triamine 1,4,7-triaza­cyclo­nonane (tacn). The threefold axis of the triamine is broken by the protonation of one of the three amine functions. The ammonium proton is bonded in an intra­molecular symmetrically bifurcated hydrogen bond to the two endodentate amine functions. Direct cation–anion contacts are established *via* N—H⋯Br hydrogen bonds between the bromide anions and tacnH^+^ cations.

## Related literature

The title compound was prepared according to a published procedure (Hay & Norman, 1979 ▶; McAuley *et al.*, 1984 ▶; Battle *et al.*, 2005 ▶) following a Richman–Atkins synthesis (Richman & Atkins, 1974 ▶). For the crystal structures of related compounds, see: Warden *et al.* (2004 ▶). A symmetrically bifurcated intra­molecular hydrogen bond to the two endodentate amine functions was also found in Me_3_tacnH^+^, see: Wieghardt *et al.* (1987 ▶).
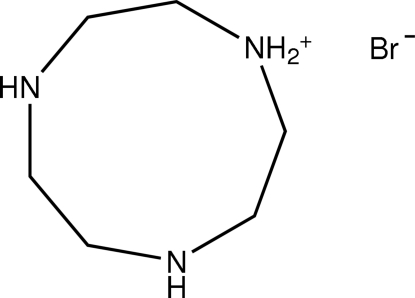


## Experimental

### 

#### Crystal data

C_6_H_16_N_3_^+^·Br^−^
*M* *_r_* = 210.12
Orthorhombic, 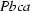

*a* = 8.11491 (18) Å
*b* = 14.1987 (4) Å
*c* = 15.3551 (4) Å
*V* = 1769.23 (8) Å^3^
*Z* = 8
Mo *K*α radiation
μ = 4.58 mm^−1^
*T* = 200 K
0.40 × 0.20 × 0.08 mm

#### Data collection

Oxford Diffraction Xcalibur diffractometer
Absorption correction: multi-scan (CrysAlisPro; Oxford Diffraction, 2009 ▶) *T*_min_ = 0.274, *T*_max_ = 0.693
12366 measured reflections
1781 independent reflections
1136 reflections with *I* > 2σ(*I*)
*R*_int_ = 0.030

#### Refinement

*R*[*F* ^2^ > 2σ(*F* ^2^)] = 0.018
*wR*(*F* ^2^) = 0.041
*S* = 0.84
1781 reflections
98 parameters
2 restraints
H atoms treated by a mixture of independent and constrained refinement
Δρ_max_ = 0.19 e Å^−3^
Δρ_min_ = −0.40 e Å^−3^

### 

Data collection: *CrysAlisPro* (Oxford Diffraction, 2009 ▶); cell refinement: *CrysAlisPro*; data reduction: *CrysAlisPro*; program(s) used to solve structure: *SIR97* (Altomare *et al.*, 1999 ▶); program(s) used to refine structure: *SHELXL97* (Sheldrick, 2008 ▶); molecular graphics: *ORTEPIII* (Burnett & Johnson, 1996 ▶); software used to prepare material for publication: *PLATON* (Spek, 2009 ▶).

## Supplementary Material

Crystal structure: contains datablocks I, global. DOI: 10.1107/S1600536809024313/zl2223sup1.cif

Structure factors: contains datablocks I. DOI: 10.1107/S1600536809024313/zl2223Isup2.hkl

Additional supplementary materials:  crystallographic information; 3D view; checkCIF report

## Figures and Tables

**Table 1:** Hydrogen-bond geometry (Å, °)

*D*—H⋯*A*	*D*—H	H⋯*A*	*D*⋯*A*	*D*—H⋯*A*
N1—H712⋯Br1^i^	0.92	2.49	3.2759 (15)	144
N1—H711⋯N2	0.92	2.28	2.726 (2)	109
N1—H711⋯N3	0.92	2.31	2.813 (2)	114
N2—H72⋯Br1^ii^	0.889 (9)	2.749 (10)	3.6123 (15)	164.1 (13)
N3—H73⋯Br1^iii^	0.871 (9)	2.661 (10)	3.4999 (16)	162.1 (16)
C2—H21⋯Br1^iv^	0.99	2.91	3.8330 (18)	156

Symmetry codes: (i) 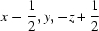; (ii) 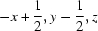; (iii) 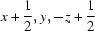; (iv) 
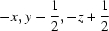
.

**Fig. 1.**
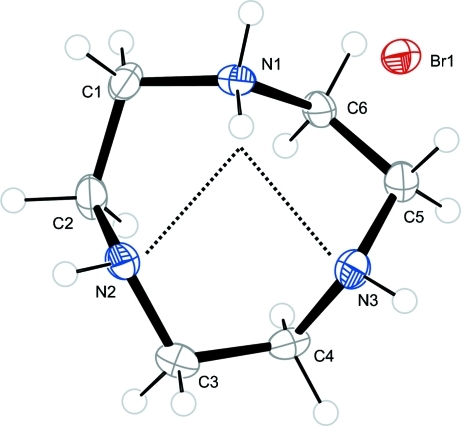
The molecular structure of the title compound, with atom labels and anisotropic displacement ellipsoids (drawn at 50% probability level) for non-H atoms. The intramolecular symmetrically bifurcated hydrogen bond is drawn as a broken line.

**Fig. 2.**
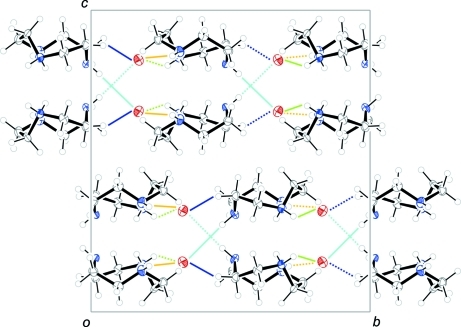
The crystalline packing of the title compound (50% probability displacement ellipsoids), viewed along [1 0 0]. Note the hydrogen-bonded chains along [001]. Color codes: N1—H···Br green, N2—H···Br blue, N3—H···Br yellow, C2—H···Br violet.

**Fig. 3.**
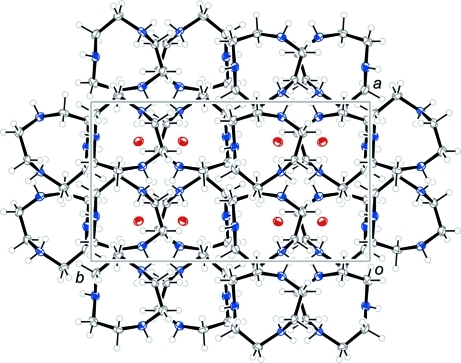
The crystalline packing of the title compound (50% probability displacement ellipsoids), viewed along [0 0 1].

## supplementary crystallographic information

### Comment

1,4,7-Triazacyclononane is a popular ligand in coordination chemistry due to its C_3_ symmetry and its propensity for facial coordination to metal ions. Although about 1500 crystallographically characterized tacn-metal complexes are known from the literature, it was not before 2005 that the crystal structure of the free parent triamine was determined (Battle *et al.*, 2005). Warden *et al.* (2004) described five salts of the threefold protonated triamine and different anions. The herein reported structure is an AB-type salt of the monoprotonated triamine and bromide counterions. The asymmetric unit contains one ion pair. Additionally, in Fig. 1 the symmetrically bifurcated intramolecular hydrogen bond to the two endodentate amine functions is shown. This bond type, also found by Wieghardt *et al.* (1987) for Me_3_tacnH^+^, accounts for the stabilization of the monoprotonation and may have contributed to the difficulty to isolate the free parent triamine.

The structure is built by bilayers that are held together by van der Waals attraction. No classical hydrogen bonds were found between the bilayers. In contrast, the bilayer itself is connected by N—H···Br hydrogen bonds which link two tacnH^+^ cations of the one layer and one tacnH^+^ cation of the adjacent layer *via* a bromide counterion. An additional C—H···Br hydrogen bond connects the tacnH^+^ cations of each monolayer with the bromide (Fig. 2).

Fig. 3 shows the packing along [0 0 1] which is more reminiscent of the structure of tacn hemihydrate (Battle *et al.*, 2005) than of the structures of the tacnH^3+^ salts which are characterized by a higher hydrogen-bond density (Warden *et al.*, 2004).

### Experimental

By following the Richman-Atkins synthesis (Richman *et al.*, 1974), 1,4,7-triazacyclononane was prepared by a method which utilizes the high degree of cyclization achieved by the reaction of the disodium salt of *N*,*N*',*N*"-tritosyldiethylenetriamine with the ditosyl derivative of 1,2-ethanediol (Hay *et al.* (1979); McAuley *et al.* (1984); Battle *et al.* (2005)). Crystals of the title compound suitable for X-ray analysis were recovered from the filtration residue of the toluene fraction which was extracted and recrystallized with acetone.

### Refinement

CH_2_ and the NH_2_ hydrogen atoms were placed in calculated positions and were included in the refinement in the riding model approximation with C—H distances of 0.99 Å and N—H distances of 0.92 Å. The positions of the amine hydrogen atoms were refined with N—H distances restrained to 0.90 (1) Å. All U_iso_(H) values were set to 1.2 *U*_eq_(C/N). The presence of pseudo-symmetry indicated by superstructure reflections along *b* suggested a higher symmetry space group *Pbcm.* Attempts to refine the structure in the space group* Pbcm* have however resulted in a disordered model with significant higher R and wR values.

### Crystal data

**Table:**

C_6_H_16_N_3_^+^·Br^−^	*F*(000) = 864
*M**_r_* = 210.12	*D*_x_ = 1.578 (1) Mg m^−^^3^
Orthorhombic, *P**b**c**a*	Mo *K*α radiation, λ = 0.71073 Å
Hall symbol: -P 2ac 2ab	Cell parameters from 4689 reflections
*a* = 8.11491 (18) Å	θ = 4.2–26.3°
*b* = 14.1987 (4) Å	µ = 4.58 mm^−^^1^
*c* = 15.3551 (4) Å	*T* = 200 K
*V* = 1769.23 (8) Å^3^	Platelet, colourless
*Z* = 8	0.40 × 0.20 × 0.08 mm

### Data collection

**Table:**

Oxford Diffraction Xcalibur diffractometer	1781 independent reflections
Radiation source: fine-focus sealed tube	1136 reflections with *I* > 2σ(*I*)
graphite	*R*_int_ = 0.030
CCD; rotation images scans	θ_max_ = 26.4°, θ_min_ = 4.7°
Absorption correction: multi-scan (*CrysAlis PRO*; Oxford Diffraction, 2009)	*h* = −10→8
*T*_min_ = 0.274, *T*_max_ = 0.693	*k* = −16→17
12366 measured reflections	*l* = −18→19

### Refinement

**Table:**

Refinement on *F*^2^	Primary atom site location: structure-invariant direct methods
Least-squares matrix: full	Secondary atom site location: difference Fourier map
*R*[*F*^2^ > 2σ(*F*^2^)] = 0.018	Hydrogen site location: inferred from neighbouring sites
*wR*(*F*^2^) = 0.041	H atoms treated by a mixture of independent and constrained refinement
*S* = 0.84	*w* = 1/[σ^2^(*F*_o_^2^) + (0.0231*P*)^2^] where *P* = (*F*_o_^2^ + 2*F*_c_^2^)/3
1781 reflections	(Δ/σ)_max_ = 0.001
98 parameters	Δρ_max_ = 0.19 e Å^−^^3^
2 restraints	Δρ_min_ = −0.39 e Å^−^^3^

### Special details

**Table:**

Experimental. CrysAlisPro (Oxford Diffraction, 2009). Empirical absorption correction using spherical harmonics, implemented in SCALE3 ABSPACK scaling algorithm.

### Fractional atomic coordinates and isotropic or equivalent isotropic displacement parameters (Å^2^)

**Table:**

	*x*	*y*	*z*	*U*_iso_*/*U*_eq_	
C1	−0.0381 (2)	0.09783 (13)	0.13984 (13)	0.0258 (5)	
H11	−0.1168	0.1100	0.0919	0.031*	
H12	−0.1018	0.0805	0.1924	0.031*	
C2	0.0771 (2)	0.01747 (13)	0.11488 (13)	0.0270 (5)	
H21	0.0168	−0.0431	0.1173	0.032*	
H22	0.1172	0.0267	0.0546	0.032*	
C3	0.3795 (2)	0.00839 (14)	0.13390 (13)	0.0263 (5)	
H31	0.3798	−0.0455	0.0930	0.032*	
H32	0.4631	−0.0043	0.1793	0.032*	
C4	0.4281 (2)	0.09747 (13)	0.08448 (12)	0.0247 (5)	
H41	0.5451	0.0927	0.0668	0.030*	
H42	0.3607	0.1023	0.0309	0.030*	
C5	0.3100 (2)	0.25835 (13)	0.09640 (13)	0.0260 (5)	
H51	0.3633	0.2770	0.0410	0.031*	
H52	0.3093	0.3140	0.1353	0.031*	
C6	0.1348 (2)	0.22841 (13)	0.07838 (12)	0.0218 (4)	
H61	0.0686	0.2840	0.0614	0.026*	
H62	0.1332	0.1831	0.0294	0.026*	
N1	0.06097 (18)	0.18365 (10)	0.15724 (9)	0.0206 (4)	
H711	0.1446	0.1683	0.1951	0.025*	
H712	−0.0052	0.2272	0.1846	0.025*	
N2	0.21724 (17)	0.01482 (11)	0.17501 (10)	0.0227 (4)	
H72	0.2032 (19)	−0.0323 (10)	0.2124 (10)	0.027*	
N3	0.40545 (19)	0.18328 (12)	0.13688 (11)	0.0249 (4)	
H73	0.4988 (15)	0.2089 (12)	0.1517 (11)	0.030*	
Br1	0.24857 (2)	0.329547 (11)	0.335223 (11)	0.02593 (7)	

### Atomic displacement parameters (Å^2^)

**Table:**

	*U*^11^	*U*^22^	*U*^33^	*U*^12^	*U*^13^	*U*^23^
C1	0.0176 (10)	0.0270 (12)	0.0329 (12)	−0.0037 (9)	0.0023 (9)	0.0063 (9)
C2	0.0288 (11)	0.0200 (12)	0.0323 (12)	−0.0069 (9)	−0.0015 (10)	0.0025 (9)
C3	0.0284 (11)	0.0241 (12)	0.0265 (12)	0.0070 (9)	0.0010 (9)	−0.0029 (10)
C4	0.0167 (10)	0.0299 (12)	0.0275 (11)	0.0039 (8)	0.0024 (9)	−0.0023 (9)
C5	0.0268 (10)	0.0208 (11)	0.0304 (12)	−0.0029 (8)	0.0041 (9)	0.0012 (10)
C6	0.0243 (10)	0.0183 (11)	0.0228 (11)	0.0017 (8)	0.0034 (9)	0.0070 (9)
N1	0.0198 (8)	0.0216 (9)	0.0204 (9)	0.0054 (7)	0.0015 (7)	0.0006 (8)
N2	0.0244 (10)	0.0186 (8)	0.0251 (9)	0.0014 (7)	0.0016 (7)	0.0061 (7)
N3	0.0201 (9)	0.0234 (10)	0.0311 (11)	−0.0039 (7)	−0.0034 (8)	−0.0020 (8)
Br1	0.02050 (10)	0.02561 (10)	0.03168 (11)	0.00302 (10)	0.00269 (11)	0.00452 (8)

### Geometric parameters (Å, °)

**Table:**

C1—N1	1.484 (2)	C4—H42	0.9900
C1—C2	1.524 (2)	C5—N3	1.457 (2)
C1—H11	0.9900	C5—C6	1.509 (3)
C1—H12	0.9900	C5—H51	0.9900
C2—N2	1.465 (2)	C5—H52	0.9900
C2—H21	0.9900	C6—N1	1.493 (2)
C2—H22	0.9900	C6—H61	0.9900
C3—N2	1.463 (2)	C6—H62	0.9900
C3—C4	1.527 (2)	N1—H711	0.9200
C3—H31	0.9900	N1—H712	0.9200
C3—H32	0.9900	N2—H72	0.889 (9)
C4—N3	1.472 (2)	N3—H73	0.871 (9)
C4—H41	0.9900		
			
N1—C1—C2	109.14 (14)	N3—C5—C6	111.89 (15)
N1—C1—H11	109.9	N3—C5—H51	109.2
C2—C1—H11	109.9	C6—C5—H51	109.2
N1—C1—H12	109.9	N3—C5—H52	109.2
C2—C1—H12	109.9	C6—C5—H52	109.2
H11—C1—H12	108.3	H51—C5—H52	107.9
N2—C2—C1	109.68 (15)	N1—C6—C5	110.44 (14)
N2—C2—H21	109.7	N1—C6—H61	109.6
C1—C2—H21	109.7	C5—C6—H61	109.6
N2—C2—H22	109.7	N1—C6—H62	109.6
C1—C2—H22	109.7	C5—C6—H62	109.6
H21—C2—H22	108.2	H61—C6—H62	108.1
N2—C3—C4	113.29 (15)	C1—N1—C6	114.89 (14)
N2—C3—H31	108.9	C1—N1—H711	108.5
C4—C3—H31	108.9	C6—N1—H711	108.5
N2—C3—H32	108.9	C1—N1—H712	108.5
C4—C3—H32	108.9	C6—N1—H712	108.5
H31—C3—H32	107.7	H711—N1—H712	107.5
N3—C4—C3	112.45 (16)	C3—N2—C2	115.35 (15)
N3—C4—H41	109.1	C3—N2—H72	110.3 (11)
C3—C4—H41	109.1	C2—N2—H72	109.1 (11)
N3—C4—H42	109.1	C5—N3—C4	116.04 (15)
C3—C4—H42	109.1	C5—N3—H73	105.5 (13)
H41—C4—H42	107.8	C4—N3—H73	112.3 (12)
			
N1—C1—C2—N2	45.5 (2)	C4—C3—N2—C2	68.6 (2)
N2—C3—C4—N3	49.5 (2)	C1—C2—N2—C3	−131.43 (16)
N3—C5—C6—N1	48.7 (2)	C6—C5—N3—C4	62.9 (2)
C2—C1—N1—C6	71.68 (19)	C3—C4—N3—C5	−128.16 (17)
C5—C6—N1—C1	−137.57 (14)		

### Hydrogen-bond geometry (Å, °)

**Table:**

*D*—H···*A*	*D*—H	H···*A*	*D*···*A*	*D*—H···*A*
N1—H712···Br1^i^	0.92	2.49	3.2759 (15)	144
N1—H711···N2	0.92	2.28	2.726 (2)	109
N1—H711···N3	0.92	2.31	2.813 (2)	114
N2—H72···Br1^ii^	0.89 (1)	2.75 (1)	3.6123 (15)	164 (1)
N3—H73···Br1^iii^	0.87 (1)	2.66 (1)	3.4999 (16)	162 (2)
C2—H21···Br1^iv^	0.99	2.91	3.8330 (18)	156

Symmetry codes: (i) *x*−1/2, *y*, −*z*+1/2; (ii) −*x*+1/2, *y*−1/2, *z*; (iii) *x*+1/2, *y*, −*z*+1/2; (iv) −*x*, *y*−1/2, −*z*+1/2.
